# Increased Expression of Retinol-Binding Protein 4 in Ovarian Endometrioma and Its Possible Role in the Pathogenesis of Endometriosis

**DOI:** 10.3390/ijms22115827

**Published:** 2021-05-29

**Authors:** Jae Chul Lee, Sung Hoon Kim, Young Sang Oh, Ju Hee Kim, Sa Ra Lee, Hee Dong Chae

**Affiliations:** Department of Obstetrics and Gynecology, University of Ulsan College of Medicine, Asan Medical Center, 88, Olympic-ro 43-gil, Songpa-gu, Seoul 05505, Korea; beas100@hanmail.net (J.C.L.); soccur@hanmail.net (Y.S.O.); xjuheex@gmail.com (J.H.K.); leesr@amc.seoul.kr (S.R.L.); hdchae@amc.seoul.kr (H.D.C.)

**Keywords:** adipokine, endometriosis, ovarian endometrioma, retinol-binding protein 4

## Abstract

Although endometriosis is a benign disease characterized by the presence of endometrial tissues outside the uterus, ectopic endometrial cells can exhibit malignant biological behaviors. Retinol-binding protein4 (RBP4) is a novel adipocyte-derived cytokine, which has important roles in regulating insulin sensitivity and energy metabolism. RBP4 is a potent modulator of gene transcription, and acts by directly controlling cell growth, invasiveness, proliferation and differentiation. Here, we evaluated the possible role of RBP4 in the pathogenesis of endometriosis. We compared the levels of RBP4 in the tissues and peritoneal fluid (PF) of women with and without endometriosis and evaluated the in vitro effects of RBP4 on the viability, invasiveness, and proliferation of endometrial stromal cells (ESCs). RBP4 levels were significantly higher in the PF of the women in the endometriosis group than in the controls. RBP4 immunoreactivity was significantly higher in the ovarian endometriomas of women with advanced stage endometriosis than those of controls. In vitro treatment with human recombinant-RBP4 significantly increased the viability, bromodeoxyuridine expression, and invasiveness of ESCs. Transfection with RBP4 siRNA significantly reduced ESC viability and invasiveness. These findings suggest that RBP4 partakes in the pathogenesis of endometriosis by increasing the viability, proliferation and invasion of endometrial cells.

## 1. Introduction

Endometriosis is a common chronic inflammatory disease that is characterized by the presence of endometrial glands and stroma outside the uterine cavity. It affects 5–10% of the women in the reproductive age and affects up to 80% of the women with pelvic pain, and 20–50% of the women with infertility [1]. The quality of life of the affected individuals is impaired due to chronic pelvic pain and other clinical symptoms, including dysmenorrhea, dyspareunia, dysuria, and dyschezia [2]. Although the pathogenesis of endometriosis has not been completely elucidated, the most popular and widely accepted etiology is Sampson’s theory of retrograde menstruation [3]. This theory describes how viable endometrial tissue can spread into the peritoneal cavity through the fallopian tubes during menstruation and elicit an inflammatory response. However, as all women with reflux menstruation do not have endometriosis, the retrograde menstruation theory alone cannot explain all cases of endometriosis.

Adipokine (or adipocytokine) is a family of cytokine that is secreted mainly from adipose tissue [4]. Although adipokines have primary roles in regulating food intake, energy balance, and homeostasis, it has been also reported that they play critical roles in various cellular functions, such as angiogenesis, pro- or anti-inflammatory actions, and the regulation of immune processes [5,6,7]. Given that the alteration of those functions of endometrial cells or other immune cells might lead to the establishment or progression of endometriosis in the pelvic cavity, several studies have therefore proposed a role for adipokines in the pathogenesis of endometriosis [8,9,10]. 

The adipokine (or adipocytokine) family comprises cytokines that are primarily secreted from adipose tissues [4]. Although the primary role of adipokines includes the regulation of food intake, energy balance, and homeostasis, it has been reported that they also play critical roles in various cellular functions, including angiogenesis, pro- and anti-inflammatory activities, and the regulation of immune processes [5,6,7]. As alterations in these functions of endometrial cells or other immune cells can induce the establishment or progression of endometriosis in the pelvic cavity, several studies have proposed a role for adipokines in the pathogenesis of endometriosis [8,9,10]. 

Retinol-binding protein 4 (RBP4) is a plasma retinol transporter that carries retinol from the liver to the periphery, and similar to adipokines, plasma RBP4 originates from adipose tissues [11]. RBP4 is a novel adipocyte-derived cytokine that plays an important role in the regulation of insulin sensitivity and energy metabolism [12]. It has been demonstrated that RBP4 partakes in the progression of insulin resistance via immune- and inflammation-related mechanisms in vascular and adipose tissues [13,14]. Furthermore, recent studies have demonstrated that certain members of cellular RBPs and the retinol signaling pathway could have a relevant role in cancer progression and in the mechanisms underlying the traits of cancer stem cells [15,16]. It has been reported that the serum levels of RBPs are significantly increased in renal cell carcinomas, suggesting their role as diagnostic and prognostic biomarkers of renal cell carcinoma [17]. RBP4 is highly expressed in ovarian cancer cells as well and a high level of RBP4 has been documented the serum samples from patients with ovarian cancer [18]. 

Based on the emerging importance of various adipokines in endometriosis, it follows that the expression of RBP4 may have a potential role in the pathogenesis of endometriosis, which is characterized by the cell growth, invasion, differentiation, migration, and activation of cytokines, chemokines, and growth factors. However, the role of RBP4 in endometriosis has not been investigated to date. Therefore, we aimed to evaluate the possible role of RBP4 in the pathogenesis of endometriosis in this study. We compared the levels of RBP4 in the peritoneal fluid (PF) and tissues of women with and without endometriosis. We also evaluated the in vitro effects of RBP4 on the viability, proliferation, and invasiveness of endometrial stromal cells (ESCs), and investigated the functional alterations in ESCs induced by the inhibition of RBP4.

## 2. Results

### 2.1. Human XL Cytokine Proteome Profile Assay

**Figure 1:**
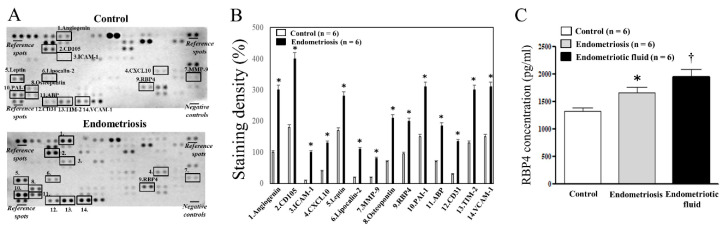
Human XL proteome profile constructed from the peritoneal fluids (PFs) of women with and without endometriosis (**A**,**B**) and RBP4 levels analyzed with ELISA assay (**C**). CD105, cluster of differentiation 105; ICAM-1, inter-cellular adhesion molecule 1; CXCL10, chemokine (C-X-C motif) ligand 10; MMP-9, matrix metallopeptidase 9; RBP4, retinol binding protein 4; PAI-1, plasminogen activator inhibitor-1; ABP, α-amino-3-hydroxy-5-methyl-4-isoxazolepropionic acid receptor binding protein; CD31, cluster of differentiation 31; TIM-2, thermal interface material 2; VCAM-1, vascular cell adhesion molecule 1. * *p* < 0.05 compared with the control group. ^†^ *p* < 0.05 compared with the control as well as the endometriosis group.

In order to identify which cytokines, chemokines, or growth factors are elevated in PF in patients with endometriosis (*n* = 6) compared to controls (*n* = 6), we compared the expression levels of 105 cytokines, chemokines, and growth factors between the two groups utilizing a human XL proteome profile array (Figure. 1A). The expression levels of angiogenin, cluster of differentiation 105 (CD105), inter-cellular adhesion molecule 1 (ICAM-1), chemokine (C-X-C motif) ligand 10 (CXCL10), leptin, lipocalin-2, matrix metallopeptidase 9 (MMP-9), osteopontin, RBP4, plasminogen activator inhibitor-1 (PAI-1), α-amino-3-hydroxy-5-methyl-4-isoxazolepropionic acid receptor binding protein (ABP), CD31, thermal interface material 2 (TIM-2) and vascular cell adhesion molecule 1 (VCAM-1) were significantly higher in the endometriosis group compared with the control group (Figure 1B, *P* < 0.05, respectively). Among those soluble proteins significantly increased in PF in patients with endometriosis compared with controls, we found that the increase in the levels of RBP4 in endometriosis has not been reported to date.

### 2.2. RBP4 Levels in the PF and Fluid from Ovarian Endometrioma

In order to verify the increase in the levels of RBP4 determined by the human XL proteome profile array, we analyzed the levels of RBP4 in the PF of women with (*n* = 6) and without (*n* = 6) endometriosis, and in the fluids from ovarian endometriomas (*n* = 6) using ELISA. The levels of RBP4 in the PF were significantly higher in the endometriosis group compared with the control group (*P* < 0.05) (Figure 1C). The levels of RBP4 in the fluids from the ovarian endometriomas were significantly higher than those in the PF of the women in the endometriosis group (*P* <0.05) and the control group (*P* < 0.05) (Figure 1C).

### 2.3. Expression of RBP4 in the Endometrium and Ovarian Endometrioma

**Figure 2:**
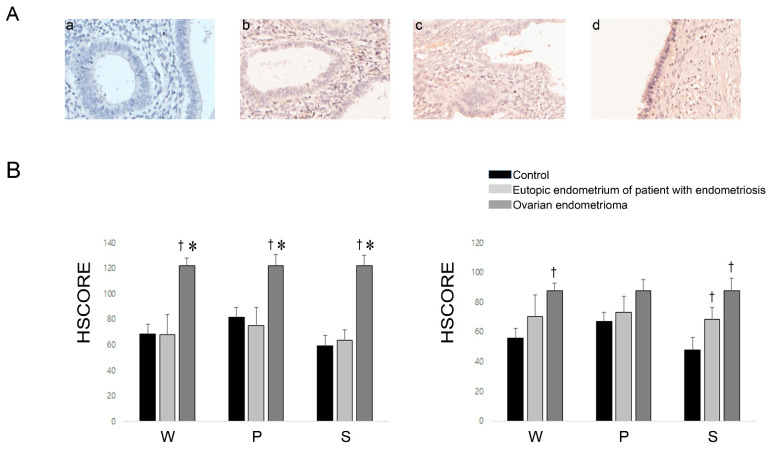
Representative micrographs of RBP4 immunohistochemistry in the eutopic endometrium of the IgG negative control (**a**), a control patient (**b**), and an endometriosis patient (**c**), and in the ovarian endometrioma (**d**) (**A**) (×200). HSCOREs of RBP4 immunostaining on the eutopic endometrium of controls and endometriosis patients as well as ovarian endometrioma in the glandular cells (left) and stromal cells (right) (**B**). W: whole menstrual stages put together; P: proliferative phase; S: secretory phase. Data of HSCOREs are expressed as mean ± SEM. ^†^ *p* < 0.05 vs. eutopic endometrium of controls, * *p* < 0.05 vs. eutopic endometrium of endometriosis patients, respectively.

RBP4 immunoreactivity was cytoplasmic and intramembranous in the endometrial glandular and stromal cells (Figure 2A). The immunoreactivity of RBP4 in the glandular cell was significantly higher in the ovarian endometrioma than in the eutopic endometrium from controls as well as patients with endometriosis during whole menstrual phases put together (*p* < 0.001; *p* < 0.001, respectively), the proliferative phase (*p* = 0.018; *p* = 0.005, respectively), and the secretory phase (*p* < 0.001; *p* < 0.001, respectively) (Figure 2B, left). The RBP4 expression was also significantly increased in the stromal cell of ovarian endometrioma compared with the eutopic endometrium from controls during whole menstrual phases put together (*p* < 0.01), and the secretory phase (*p* = 0.002) (Figure 2B, right). The immunoreactivity of RBP4 in the stromal cell was significantly higher in the eutopic endometrium of the patients with endometriosis compared with that of the controls only during the secretory phase (*p* = 0.012) (Figure 2B, right).

### 2.4. Cell Viability Assay after RBP4 siRNA Transfection and Human Recombinant-RBP4 (HR-RBP4) Treatment 

**Figure 3:**
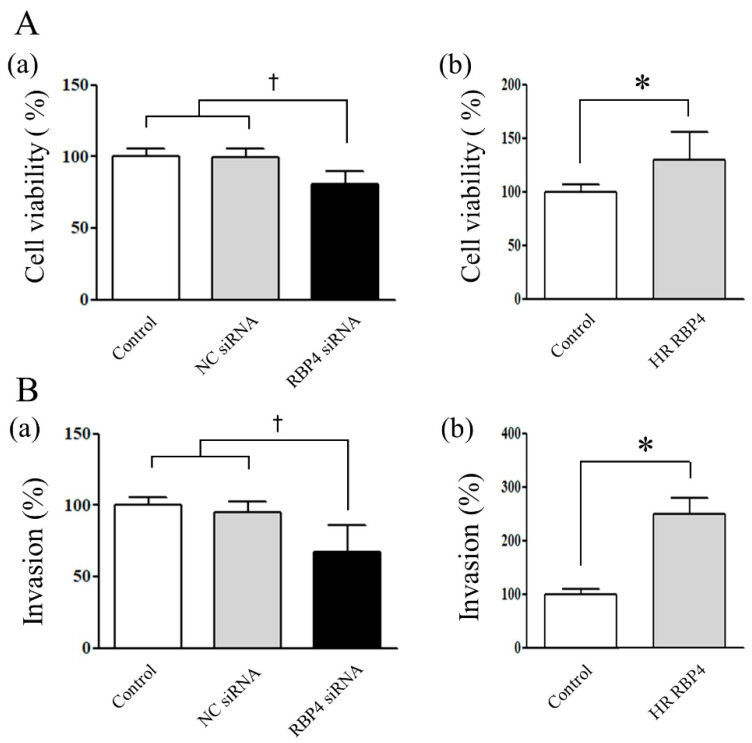
Cellular viability (**A**) and invasiveness (**B**) of endometrial stromal cells following treatment with the vehicle, transfection with the negative control siRNA or RBP4 siRNA (**a**), or treatment with HR-RBP4 (**b**). The error bars represent the mean ± SEM. Data are expressed as a percentage, in which the cells treated with the control are normalized to100%. ^†^ *p* < 0.05 compared with vehicle group as well as negative control siRNA transfection group.* *p* < 0.05 compared with the control group. NC: negative control.

In order to investigate the effect of RBP4 on the viability of the ESCs, we first transfected the ESCs with the negative control siRNA and RBP4 siRNA. The viability of the ESCs significantly decreased after 48 h of transfection with RBP4 siRNA compared to that of the ESCs treated with the vehicle (*p* < 0.05) or transfected with the negative control siRNA (*p* < 0.05) (Figure 3A(a)). We subsequently treated the ESCs with the vehicle or HR-RBP4, and compared the viability of the ESCs. The viability of the ESCs treated with HR-RBP4 was significantly increased compared to that of the control at 48 h (*p* < 0.05) (Figure 3A(b)).

### 2.5. Invasion Assay after RBP4 siRNA Transfection and Human Recombinant-RBP4 (HR-RBP4) Treatment 

In order to investigate the effect of RBP4 on the invasiveness of the ESCs, we first transfected the ESCs with the negative control siRNA and RBP4 siRNA. The invasiveness of the ESCs significantly decreased after 48 h of transfection with RBP4 siRNA, compared with that of the ESCs that were treated with the vehicle (*p* < 0.05) or transfected with the negative control siRNA (*p* < 0.05) (Figure 3B(a)). We subsequently treated the ESCs with the vehicle or HR-RBP4, and compared the invasiveness of the ESCs. The invasiveness of the ESCs treated with HR-RBP4 was significantly increased in comparison to that of the control samples at 48 h (*p* < 0.05) (Figure 3B(b)).

### 2.6. Expression of Matrix Metalloproteinases (MMP)-2 and MMP-9 Following RBP4 siRNA Transfection and Treatment with HR-RBP4 

**Figure 4:**
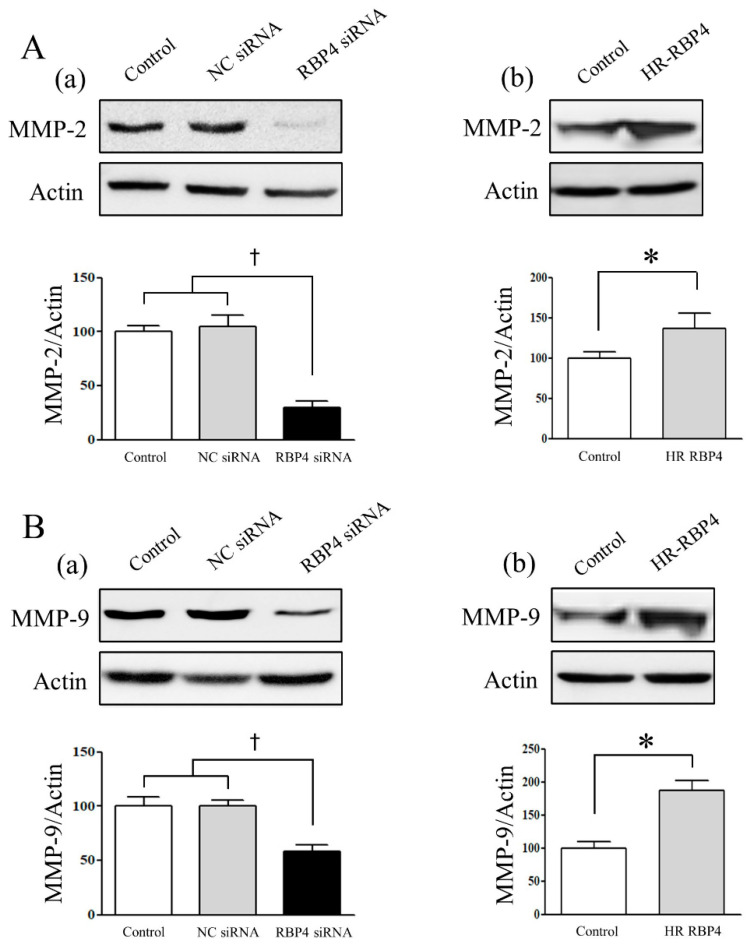
Expression of MMP-2 (**A**) and MMP-9 (**B**) in the endometrial stromal cells following treatment with the vehicle, transfection with the negative control siRNA or RBP4 siRNA (**a**), or treatment with HR-RBP4 (**b**). The error bars represent the mean ± SEM. Data are expressed as a percentage, in which the cells treated with the control are normalized to 100%, ^†^ *p* < 0.05 compared with vehicle group as well as negative control siRNA transfection group. * *p* < 0.05 compared with the control group. NC: negative control.

The expression of MMP-2 and MMP-9 in the ESCs was analyzed following treatment with the vehicle, transfection with the negative control siRNA or RBP4 siRNA, or treatment with HR-RBP4. The expression of MMP-2 and MMP-9 in the ESCs transfected with RBP4 siRNA significantly decreased compared with that in the ESCs treated with the vehicle (*p* < 0.05) or transfected with the negative control siRNA (*p* < 0.05) after 48 h (Figure 4A(a),B(a)). When the ESCs were treated with the vehicle or HR-RBP4, the expression of MMP-2 and MMP-9 in the ESCs treated with HR-RBP4 significantly increased compared with that in the controls at 48 h (*p* < 0.05 and *p* < 0.05, respectively) (Figure 4A(b),B(b)).

### 2.7. Bromodeoxyuridine (BrdU) Assay Following RBP4 siRNA Transfection and Treatment with HR-RBP4

**Figure 5:**
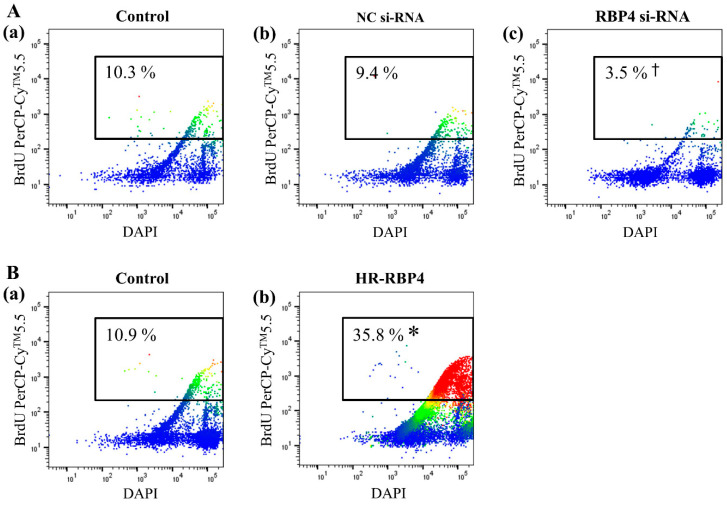
Cellular proliferation analyzed using flow cytometry with BrdU staining in endometrial stromal cells following treatment with the vehicle (**a**), transfection with the negative control siRNA (**b**) or RBP4 siRNA (**c**) (**A**), or treatment with HR-RBP4 (**Bb**). ^†^ *p* < 0.05 compared with vehicle group (**Aa**) as well as negative control siRNA transfection group (**Ab**). * *p* < 0.05 compared with the control group (**Ba**). NC: negative control.

In order to investigate the effect of RBP4 on cellular proliferation, the BrdU assay was performed in the ESCs following treatment with the vehicle, transfection with the negative control siRNA or RBP4 siRNA, or treatment with HR-RBP4. The percentage of BrdU-labeled cells was calculated as the ratio of the levels of the BrdU-positive cells (BrdU+ gate) to those of all the cells. The population of BrdU-positive cells significantly decreased after 48 h of transfection with RBP4 siRNA (3.5%) (Figure 5A(c)) compared with that of the vehicle-treated group (10.3%) (*p* < 0.05) (Figure 5A(a)) or the group transfected with the negative control siRNA (9.4%) (*p* < 0.05) (Figure 5A(b)). The population of BrdU-positive cells increased after 48 h of treatment with HR-RBP4 (35.8%) (Figure 5B(b)), compared with that of the control group (10.9%) (*p* < 0.05) (Figure 5B(a)). 

## 3. Discussion

Originally known as a retinol-transporter secreted from the liver, RBP4 is also produced from adipose tissues, and the serum RBP4 levels have been shown to be elevated in obese, insulin-resistant mice and humans [19,20]. It has been demonstrated that RBP4 activates macrophages and induces the release of pro-inflammatory cytokine, which leads to the blockade of insulin signaling in adipose tissues [21]. RBP4 can also activate the pro-inflammatory response via the c-Jun N-terminal protein kinase 1/2 or Toll-like receptor pathways [22]. RBP4 can increase the proliferation of vascular smooth cells via several signaling pathways [23], and upregulates the proliferation and invasion of trophoblastic cells [24]. It has been demonstrated that the serum levels of RBP4 are elevated in patients with ovarian cancer, and the expression of RBP4 is increased by approximately four-fold in ovarian cancer tissues compared with its expression in the benign ovarian tissues [18,25]. The overexpression of RBP4 leads to the increased migration and proliferation of ovarian cancer cells via the induction of MMP 2/9, which is inhibited by RBP4 knockdown [25]. It is suggested that RBP4 functions as a classical cytokine and activates cell signaling pathways transduced by its cognate receptor, vitamin A receptor, following stimulation by retinoic acid 6 (STRA6) [26].

Numerous theories have been proposed in relation to the etiological factors of endometriosis, and it has been well established that endometriosis is an estrogen-dependent disease with chronic inflammation, that is characterized by immune activation, inflammation, altered immune response, and oxidative stress. We therefore hypothesized that high levels of cytokines, chemokines, and growth factors can promote the invasion, migration, differentiation, and proliferation of endometrial cells, which can result in the establishment of endometriosis outside the uterus. We first analyzed the relative expression levels of 105 soluble human proteins in the PF of women with or without endometriosis using the human XL proteome profile array kit. The levels of angiogenin, CD105, ICAM-1, CXCL10, leptin, lipocalin-2, MMP-9, osteopontin, RBP4, PAI-1, ABP, CD31, TIM-2, and VCAM-1 were higher in the endometriosis group than in the control group. These proteins partake in numerous biological processes, including cellular growth, differentiation, gene expression, migration, immunity, and inflammation. Among those soluble proteins shown to be higher in the endometriosis patients, the increase in the levels of RBP4 in endometriosis has not been reported to date. Therefore, the present study investigated whether the expression of RBP4 increases in endometriotic tissues and evaluated the functional alterations induced by the expression of RBP4 in ESCs.

In order to validate the human XL proteome profile array data, we investigated whether the levels of RBP4 are elevated in the PF or endometriotic fluid collected from the ovarian endometriomas of patients with endometriosis, in comparison with the levels in the control samples. The results confirmed that the levels of RBP4 were evidently higher in the PF and endometriotic fluid collected from the ovarian endometriomas of patients with endometriosis. We subsequently evaluated whether the expression of RBP4 is increased in endometriotic tissues in comparison with that in the control samples, and compared the immunoreactivity of RBP4 among the three groups, eutopic endometrium of the controls, eutopic endometrium of endometriosis patients, and ovarian endometrioma. We observed that the expression of RBP4 in the glandular and stromal cells was evidently higher in the ovarian endometrioma group than in the control group, which strongly suggested that the increase in the levels of RBP4 in the PF or endometriotic fluid results from the increased expression of RBP4 in ovarian endometriomas.

Based on the marked increase in the expression of RBP4 in the ovarian endometrioma group, we investigated the functional alterations in the ESCs induced by RBP4 siRNA transfection or treatment with HR-RBP4. The results demonstrated that both cellular viability and proliferation significantly decreased following RBP4 siRNA transfection, and significantly increased following treatment with HR-RBP4, which was consistent with the results of the cell viability assay and BrdU assay. The cellular invasiveness of the ESCs was also markedly reduced following RBP4 siRNA transfection, and increased by treatment with HR-RBP4, which was consistently supported by the MMP-2/9 expression data in the present study. These findings strongly suggest that RBP4 may partake in the pathogenesis and/or pathophysiology of endometriosis by increasing the viability, proliferation, and invasiveness of endometrial cells.

To the best of our knowledge, this study is the first to report the possible role of RBP4 in the pathogenesis and/or pathophysiology of endometriosis. However, several studies have previously suggested that various adipokines may play a role in the pathogenesis of endometriosis. Leptin, a product of the obese (ob) gene, was shown to be markedly elevated in the serum and PF of patients with endometriosis [27], and it has been reported that the expression of leptin and its receptor increases in ovarian endometriomas [9]. Leptin promoted the migration and invasion of human endometriotic cells via the upregulation of MMP-2, which is regulated by the Janus kinase 2/signal transducer and activator of transcription 3 (JAK2/STAT3) signaling pathway [8]. It has been demonstrated that resistin, a member of the adipokine family, is also elevated in the PF of patients with endometriosis [10], and that the expression of resistin significantly increases in the ectopic endometrial tissues of patients with endometriosis [28].

Given that the prevalence of endometriosis is inversely associated with body mass index (BMI) or obesity [29], the cause underlying the marked increase in the concentration and expression of these adipokines in the PF and endometriotic tissues remains to be elucidated. The results of the present study are consistent with those of previous reports, which demonstrated that the BMI of the endometriosis group was significantly lower than that of the control group [10,28]. It might be possible that the expression levels of RBP4 are higher in the endometriotic tissues, as observed in other cancer tissues [15,16,17,18,19]. The increased expression of RBP4 may lead to the progression of endometriosis via the activation of pro-inflammatory processes, cellular proliferation, and invasiveness. Despite the absence of relevant data, it is also possible that visceral omental adipose tissues produce more RBP4 in patients with endometriosis irrespective of their BMI, which might lead to increased levels of RBP4 in the PF and increase the expression of RBP4 in endometriotic tissues via a paracrine effect.

Considering that RBP4 serves as a signaling molecule activating downstream pro-oncogenic signaling pathways after binding to the membrane receptor STRA6 [26], the data of the present study seem to support the idea that ectopic endometrial cells can exhibit malignant biological behaviors in endometriosis. Indeed, the overexpression of STRA6 has been reported in human malignancies along with oncogenic transformation driven by holo-RBP and STRA6 [15,16]. However, it was demonstrated that the expression of STRA6 is decreased in endometriotic tissues, which leads to decreased retinol uptake and reduced hydroxysteroid 17-β dehydrogenase that converts estradiol to estrone [30,31]. These studies suggested that decreased retinol uptake, metabolism, and action partake in the pathogenesis of endometriosis. In this study, we could not evaluate whether the increased expression of RBP4 had any effects on STRA6 expression, retinol uptake and metabolism, or estradiol synthesis in endometrial cells. Further studies are necessary to clarify the mechanisms in which both the increased RBP4 levels and the aberrant retinoid signaling pathways are involved in the pathogenesis or pathophysiology of endometriosis. 

In this study, it was not possible to clarify whether the increased RBP4 level or expression itself can play a crucial role in the establishment of endometriosis or may have resulted from disease progression due to the activation of pro-inflammatory processes, endometrial proliferation, or invasion after the initial establishment of endometriotic lesions. All the women in the control group were not disease-free, and had benign gynecological diseases other than endometriosis. We could not explain the main origin of the increased of RBP4 levels in the patients with endometriosis, which is well known to be inversely associated with BMI or fat mass. Despite these limitations, to our knowledge, this study is the first to demonstrate a marked elevation in the levels of RBP4 in the PF of women with endometriosis as well as a significant increase in the expression of RBP4 in ovarian endometriomas, and to elucidate the functional relevance of RBP4 in the development of endometriosis. Further well-designed study is necessary to overcome the limitations of the present study.

## 4. Materials and Methods 

### 4.1. Patients, Collection, and Preparation of Tissues and Samples

The PF was collected in the follicular phase from patients with advanced stage endometriosis (*n* = 6) and from the control group (*n* = 6) during laparoscopic surgery. The PF was collected from the posterior cul-de-sac or utero-vesical pouch via a laparoscopic cannula. The samples of PF with blood contamination were excluded. The fluids from the ovarian endometrioma were also obtained from patients with advanced stage endometriosis (*n* = 6) during laparoscopic surgery using a laparoscopic needle. The cellular components of the PF and endometriotic fluids were removed by centrifugation at 3500 revolutions per minute for 15 min. The supernatants were subsequently collected and stored in aliquots at −70 to −80 °C until further analyses. 

For immunohistochemical staining, we used the sections of endometria and ovarian endometriomas from our previous study [32]. Briefly, the endometrial sections were obtained from a total of 34 women with histological evidence of endometriosis who had undergone hysterectomy and those from 38 women with carcinoma in situ of the uterine cervix served as controls. Sections of ovarian endometrioma were also obtained from 25 nulliparous women who had undergone ovarian cystectomy due to ovarian endometrioma. In the endometriosis group, the extent of the disease was staged according to the guidelines of the American Society for Reproductive Medicine [33]. The BMI was significantly higher in the control group (22.9 ± 0.3 kg/m^2^) than in the endometriosis–hysterectomy group (21.5 ± 0.2, *p* < 0.05) and the ovarian endometrioma group (20.9 ± 0.3, *p* < 0.05). 

For the ESC cultures, endometrial samples were obtained from fertile women diagnosed with intramural leiomyoma at the time of hysterectomy, who had no evidence of endometrial abnormalities, adenomyosis, or pelvic endometriosis, and who had not received any hormonal medications in the preceding 3 months. The endometrial samples were placed in Hanks’ balanced salt solution and transported to the laboratory for the isolation and culture of ESCs. Written informed consent was obtained from each patient using consent forms and protocols approved by the Institutional Review Board for Human Research of Asan Medical Center. 

### 4.2. Human XL Proteome Profile Assay

A human XL proteome profile array was initially performed for analyzing the PF, according to manufacturer’s instructions (ARY022B, R&D Systems, Minneapolis, MN, USA), for identifying the cytokines, chemokines, and growth factors that are elevated in the PF of patients with endometriosis, in comparison to that of the control. The blotting of the PF samples (*n* = 6 per group) was performed separately in duplicate, and the average of these two-pixel densities was used to calculate the average density using Image J software. The background staining and spot sizes were analyzed according to the manufacturer’s instructions. The images were converted to 8-bit inverted jpeg files and the spots were encircled. Equal spot sizes were analyzed for each blot.

### 4.3. Immunohistochemistry

For the immunohistochemical studies, 3-μm-thick sections were cut from formalin-fixed, paraffin-embedded tissue blocks of endometriotic lesions and mounted on glass slides. The immunohistochemical analyses were performed using an automated Benchmark XT slide preparation system (Ventana Medical systems, Inc., Oro Valley, AZ, USA). Deparaffinization, epitope retrieval, and immunostaining were performed according to the manufacturer’s instructions by using cell-conditioning solutions (CC1) and the BMK ultraVIEW diaminobenzidine detection system (Ventana Medical Systems). The sections of the lesions were stained with RBP4 (1:500; Abcam). The positive signals were amplified using ultra-VIEW copper, and the sections were counterstained with hematoxylin and DAB. Images of the slides were captured using a Vectr intelligent Slide Analysis System (version 2.0.8, PerkinElmer Inc., Waltham, MA, USA). The images were subsequently used to train the Form Advanced Image Analysis Software (version 2.2, PerkinElmer Inc.) for quantitative image analysis.

### 4.4. Isolation and Culture of ESCs

The ESCs were separated and maintained in a monolayer culture as previously described [32]. Following isolation, the ESCs were subjected to standard methods of trypsinization, plated in culture dishes, and grown in DMEM supplemented with 10% charcoal-stripped calf serum (Flow Laboratories, Sunnyvale, CA, USA). The ESCs obtained after the first passage were immunocytochemically assayed using specific cell surface markers. We have previously demonstrated that the purity of the isolated ESCs is greater than 95%. We only used the cells obtained after the first passage in all the experiments with ESCs.

### 4.5. Experimental Setup

Each of the experiments on the ESCs was performed using cells separately prepared from the endometrial tissue specimens obtained from different patients. When the culture had reached a confluence of 70%, the ESCs were treated with serum-free, phenol red-free media (Sigma-Aldrich, St. Louis, MO, USA) for 48 h before treatment. In order to evaluate the effect of HR-RBP4 (catalog no. 3378-LC, R&D Systems) and RBP4 siRNA on cellular viability, invasiveness, the expression of MMP-2/9, and proliferation, the cultures were separately treated with the vehicle (control), 25 ng/mL HR-RBP4, 30 nmol/L negative control siRNA, or 30 nmol/L RBP4 siRNA for 48 h.

### 4.6. Cell Viability Assay

Cell viability was assessed using an EZ-Cytox cell viability assay kit (Daeillab Service, Seoul, Korea). The ESCs transfected with RBP4 siRNA or the negative control siRNA were incubated for 48 h (*n* = 4). The cells were subsequently detached and seeded on 96-well plates at a density of 5000 cells/well. Then, 10 μL of the highly sensitive, water soluble tetrazolium salt assay reagent was added to each well on day 2 for the ESCs. The plates were incubated for 1 h at 37 °C. Cell viability was determined by measuring the absorbance of the samples at 450 nm.

### 4.7. Invasion Assay

Invasion assays were performed in a 96-well trans-well containing inserts with a pore size of 8 μm (Corning, Corning, New York, NY, USA), and coated with Cultrex Basement Membrane Extract (Trevigen, Gaithersburg, Maryland). The ESCs transfected with RBP4 siRNA or the negative control siRNA were incubated for 48 h (*n* = 4). The cells were then starved in serum-free DMEM for 24 h. Then, 50 μL of cell suspension (50,000 cells/well) was added to the top compartment and 100 μL of serum-free DMEM was added to the bottom compartment of the chamber. The chambers were incubated at 37 °C in humidified air containing 5% CO_2_ for 24 h. Both the compartments of the chambers were subsequently washed with washing buffer, and the cells that invaded the bottom chamber were labeled by incubating with 5 μg/mL calcein-AM (Trevigen, Gaithersburg, MD, USA) in cell dissociation solution for 1 h at 37 °C. Cell invasion was determined by measuring the absorbance of the samples at excitation and emission wavelengths of 485 nm and 520 nm, respectively, with the same parameters (time and gain), using a Victor X3 multilabel plate reader (PerkinElmer Inc.).

### 4.8. Protein Extraction and Western Blot Analysis

The cells were resuspended in lysis buffer (Cell Signaling Technology, Danvers, MA, USA) containing a protease inhibitor cocktail (complete mini tablet, Roche) (*n* = 4). The membranes were blocked by incubation for 1 h at room temperature, and they were subsequently incubated over-night with the primary antibodies against MMP-2 (Cell Signaling Technology), MMP-9 (Cell Signaling Technology), and actin (Cell Signaling Technology) at 4 °C. The membranes were washed thrice and subsequently incubated with a secondary horseradish peroxidase-conjugated anti-IgG antibody (Invitrogen, Carlsbad, CA, USA) and visualized using a Pierce chemiluminescent substrate (Life Technologies, Carlsbad, CA, USA). The densitometric quantification of the bands was performed using a laser densitometer. The bars represent the mean of data obtained from three replicates.

### 4.9. Proliferation Assays

The ESCs were seeded onto 6-well culture plates at a density of 2 × 10^5^ cells/well for 24 h and treated with HR-RBP4 and the negative control siRNA for 48 h (*n* = 4). The cellular proliferation in these cultures was measured by flow cytometry (FACS Canto II cytometer, Becton Dickinson, Mountainview, CA, USA) using a Cell Proliferation Kit (BD Pharmingen, 562253), according to the manufacturer’s protocol. The assays were performed in triplicate and the results represent the data obtained from three independent experiments.

### 4.10. Statistical Analysis

All the data were analyzed by the Kolmogorov–Smirnov test to evaluate whether they followed a normal distribution. When the data followed a normal distribution, continuous variables were compared using Student’s t test (two groups) or analysis of variance and Fisher’s least significant difference post hoc test for pairwise comparisons (three groups). The Mann–Whitney U test was used for comparing the data (two groups) when the data did not follow a normal distribution. All the statistical computations were performed using the Statistical Program for the Social Sciences software, version 14.0. *P* < 0.05 was considered to be statistically significant. 

## 5. Conclusions

The results of this study strongly suggest that RBP4 might partake in the pathogenesis and/or pathophysiology of endometriosis by increasing the viability, proliferation, and invasiveness of endometrial cells.

## Data Availability

The excel data used to support the findings of this study were supplied by Sung Hoon Kim under license, and requests for access to these data should be made to S.H.K.
